# Overview of distinct 5-methylcytosine profiles of messenger RNA in normal and knock-down NSUN2 colorectal cancer cells

**DOI:** 10.3389/fgene.2023.1121063

**Published:** 2023-04-24

**Authors:** Yu Lin, Zhifang Zhao, Wenqiang Nie, Manting Huang, Jiazhong Cai, Yadong Wang, Hesong Wang, Yongmei Huang, Yang Bai

**Affiliations:** ^1^Department Guangdong Provincial Key Laboratory of Gastroenterology, Department of Gastroenterology, Institute of Gastroenterology of Guangdong Province, Nan Fang Hospital, Southern Medical University, Guangzhou, China; ^2^Department of Gastroenterology, Baiyun Branch, Nan Fang Hospital, Southern Medical University, Guangzhou, China; ^3^ Science and Technology Innovation Center, Guangzhou University of Chinese Medicine, Guangzhou, China; ^4^Department of Traditional Chinese Medicine, The First Affiliated Hospital of Guangdong Pharmaceutical University, Guangzhou, China; ^5^Department of Vascular Intervention, Zhongshan Hospital of Traditional Chinese Medicine, Affiliated to Guangzhou University of Chinese Medicine, Zhongshan, China

**Keywords:** 5-methylcytosine, colorectal cancer, RNA methylation, MeRIP-seq, NSun2

## Abstract

**Background:** Colorectal cancer (CRC) is a harmful cancer with high morbidity and poor prognosis. There is growing evidence that RNA methylation is closely related to the occurrence of cancer and its malignant biological behavior. N6-methyladenosine (m^6^A) methylation is the most common RNA modification in eukaryotes, and its multiple regulatory mechanisms in CRC have been elucidated from multiple perspectives. At the same time, the role of 5-methylcytosine (m5C), another important and widely distributed methylation modification, in CRC is far from being elucidated.

**Methods:** In this study, we used RNA immunoprecipitation sequencing combined with bioinformatics methods to identify the m5C peaks on messenger RNA (mRNA) in HCT15 cells and sh-NSUN2 HCT15 cells, understand which transcripts are modified by m5C, and characterize the distribution of m5C modifications. In addition, we performed further bioinformatics analysis of the detected data to initially clarify the potential function of these m5C-modified transcripts.

**Results:** We found significant differences in the distribution of m5C between HCT15 cells and sh-NSUN2 HCT15 cells, suggesting that m5C is likely to play a key role in the occurrence and development of CRC. Furthermore, Gene Ontology (GO) enrichment analysis showed that genes altered by m5C were mainly enriched in phylogeny, synaptic membrane, and transcription factor binding. The Kyoto Encyclopedia of Genes and Genomes (KEGG)pathway analysis showed that the genes altered by m5C are enriched in ECM receptor interaction pathway, the circadian pathway, and the cAMP signaling pathway.

**Conclusion:** Here, our study preliminarily revealed the different distribution patterns of m5C between HCT15 cell and sh-NSUN2 HCT15 cell. Our results open a new window to understand the role of m5C RNA methylation of mRNA in the development of CRC.

## 1 Introduction

Colorectal cancer (CRC) is the third most common cancer after breast and lung cancer, with more than 1.9 million new cases each year (Masdor et al., 2022). In China, the incidence and mortality of colon cancer have been on the rise in recent years, ranking among the top five tumors in China, the diagnosis is often in the middle and advanced stages, the prognosis is poor, and distant metastasis is the leading cause of death in patients with colorectal cancer. With the continuous improvement of medical level, surgery, radiotherapy and chemotherapy, targeted therapy, immunotherapy, and other comprehensive treatment measures have continuously improved the overall survival (OS) of colorectal cancer patients, but its overall efficacy is still poor, metastatic colorectal cancer (mCRC). The 5-year survival rate is only about 14% (Siegel et al., 2019). Therefore, exploring the mechanism of colorectal cancer occurrence and development and finding effective targets for tumor treatment are crucial for the prevention and treatment of colorectal cancer. With the rise of epigenetics research, more and more scholars are trying to understand the pathogenesis of colorectal cancer from the perspective of epigenetics.

As a Frontier and hot spot in the field of epigenetics, RNA posttranscriptional modification has received more and more attention in recent years. Of the more than 170 RNA post-transcriptional modifications found so far, 2/3 are methylation modifications, including 5-methlcytosine (m^5^C), N6-methyadenosine (m^6^A), N1-methyladenosine (m^1^A), N7-methylguanosine (m^7^G), 2′-O-methylribonucleosides (2′-OMe-NRs), *etc.* The m5C methylation modification of RNA refers to methylation on the fifth carbon atom on RNA cytosine, which was first discovered in rRNA and later on transporter RNA (tRNA), messenger RNA (mRNA), and long non-coding RNA (lncRNA) (Trixl and Lusser, 2019). m5C modifications on RNA are widespread and play an important role in RNA stability, translation and subcellular localization. m5C modification is closely related to the activation of proto-oncogenes, and there is a large difference in the expression of the key methyltransferase NSUN2 modified by m5C in cancerous and adjacent tissues. The m5C modification of RNA is dynamically regulated by m5C methyltransferase and m5C demethylation transferases, and RNA is modified by m5C under the catalysis of m5C methyltransferase, and this m The m5C modification can be erased by the demethylase enzyme of m5C. RNA modified by m5C binds to specific recognition proteins and exerts action Specific biological functions (Trixl and Lusser, 2019). The main methyltransferases modified by m5C include NSUN1-7 and DNMT2, which share the common characteristics of conserved cysteine residues that catalyze m5C methylation of multiple types of RNA with the help of methylated donor S-adenosyl-L-methionine (SAM) (Xue et al., 2020).

NSUN family proteins are m5C methyltransferases that have been widely studied in recent years. Human NSUN family proteins have seven members, all have m5C methyl transfer functional domains, and contain two cysteine necessary to catalyze methyl transfer function. The common mechanism of the NSUN family catalyzing methyl transfer is that the covalent binding occurs between the cysteine of methyltransferase and the cytosine of RNA to form a covalent intermediate, and then the electron-rich cytosine ring performs nucleophilic addition to the methyl group on SAM, and finally completes methylation. The most widely studied in the NSUN family is NSUN2, NSUN2 is mainly localized in the nucleus and plays an important role in cell differentiation and tumorigenesis. There is now evidence that NSUN2 can catalyze m5C methylation of many types of RNA, including tRNA, mRNA, and non-coding RNA. Dnmt2 is a methyltransferase of tRNA and micro RNA, located primarily in the nucleus. Knockout of ALKBH1 in 293T cells can lead to a decrease in f5C modifications on mitochondrial tRNA, which in turn leads to impaired mitochondrial translation and respiratory function (Kawarada et al., 2017). The m5C methylation recognition proteins reported so far mainly include ALYREF which regulates nucleation, RAD52 that is involved in DNA damage repair, and YBX1 that regulates RNA stability (Yang et al., 2017; Yang et al., 2019). M5C methylation and its modifiers play a key role in the development of cancers. Several studies have shown that NSUN2 is abnormally high in gastric and colorectal cancers, which is closely related to poor prognosis (Okamoto et al., 2012). Overexpression of NSUN2 promotes proliferation and migration of tumor cells and knocking down NSUN2 inhibits these processes (Manning et al., 2020). Further studies found that the tumor suppressor gene P57, as a downstream target of NSUN2, affected the proliferation ability of tumor cells. In cells, the expression of P57 and NSUN2 were negatively correlated, and the high expression of NSUN2 reduced the stability of mRNA, inhibited the expression of P57, and promoted the proliferation of tumor cells by catalyzing m5C methylation in the 3′UTR region of P57 mRNA (Mei et al., 2020). NSUN5 is significantly more expressed in colorectal cancer, and studies have shown that knocking out NSUN5 in colorectal cancer cell lines causes cell cycle blockade by inhibiting cyclin-dependent kinase (CDKs) signaling, significantly inhibiting cell proliferation (Jiang et al., 2020).

Methylated RNA immunoprecipitation sequencing (MeRIP-seq) is a commonly used m5C modification detection technology. This method breaks the purified total RNA or mRNA into 100–150 nt RNA fragments; Subsequently, the RNA fragments were co incubated with m5C modified antibody, and the RNA fragments containing m5C modification were enriched by antibody for library building and sequencing. Finally, the m5C modified transcriptome region was obtained by comparing with the control group library co incubated with m5C modified antibody. With advances in MeRIP-seq, researchers have been able to characterize RNA methylation sites precise (García-Vílchez et al., 2019).

In this study, we constructed HCT15 cell lines with NSUN2 knockdown, performed MeRIP-seq on HCT15 cells with NSUN2 knockdown and normal HCT15 cells, and performed m5C-specific analysis and deep bioinformatics analysis on m5C mRNA. The results showed that there were remarkable differences in the number and distribution of m5C between the two groups. The number of m5C methylation peak in normal HCT15 cells was much higher than that in NSUN2 knockdown cells, and the distribution of m5C methylation peak was significantly different, involving all chromosomes. Further bioinformatics analysis showed that the two groups with different degrees of m5C methylation could cause significant different changes in cell function. Our study preliminarily suggests a possible association between CRC and m5C modification in mRNA and predict potential functional changes resulting from the difference in m5C modification in mRNA. All these will provide a solid basis for further understanding of the pathogenesis of CRC from the perspective of m5C modification.

## 2 Materials and methods

### 2.1 Establishment of stable knockdown cell line

The NSUN2 knockdown lentivirus construction system was obtained from Obio Company (Shanghai, China). HCT15 cells were stably transfected with NSUN2 knockdown lentivirus (shNUSN2) and a corresponding control (shNC) with an anti-puromycin plasmid. The stably transfected HCT15 cell line was treated with puromycin (4 μg/mL) for 7 days for selection. The sequences of shRNA is CAC​GTG​TTC​ACT​AAA​CCC​TAT.

### 2.2 RNA extraction and fragmentation

We extracted total RNA using Trizol reagent (Invitrogen, California, United States) following the manufacturer’s instructions. Use the Ribo-Zero rRNA Removal Kit (Illumina, Inc., CA, United States) to reduce rRNA content. The quality of RNA is assessed using its OD260/OD280 ratio, which is a measure of nucleotide-to-protein ratio based on optical density measured using spectrophotometry. RNA purity with OD260/OD280 values in the range of 1.8–2.1 is considered acceptable, and RNA extracted from all samples meets this standard.

### 2.3 Library construction and sequencing

Methylated RNA immunoprecipitation sequencing (MeRIP-seq) is performed based on the previously reported procedure (Luo et al., 2019). Total RNA was cleaved into 100 base pair fragments using the GenSeqTM m5C RNA IP Kit (GenSeq Inc., China), followed by m5C immunoprecipitation using the NEBNext^®^ Ultra II Directed RNA Library Preparation Kit (New England Biolaboratories, United States). The cDNA library was evaluated using the Bioanalyzer 2,100 System (Agilent Technologies United States) and the library was sequenced using an Illumina Hiseq instrument with 150 bp pairing readings. The GenSeq m5C MeRIP kit has been designed specifically for RNA m5C studies. The MeRIP method enables specific enrichment of m5C modified regions in the transcriptome. This kit contains all the core reagents in the MeRIP process, such as proven m5C antibodies, RNA fragmentation reagents, protein A/G beads, IP and elution buffers, providing a better cost performance. In addition, the kit contains control IgG antibody, which is convenient for users to conduct experimental quality control and ensure the rigor of the experiment. The optimized experimental procedure is extremely simple and efficient, and the enriched m5C RNA can be widely used in subsequent quantitative RT-PCR, high-throughput sequencing and other detection and analysis. Its official website is http://www.cloud-seq.com.cn/case-item-353.html.

### 2.4 Identifications and analysis of 5-methylcytosine peaks

Pairing reads were quality controlled using a quality criterion of 1/1000 (Q30) error base call probability in the Illumina HiSeq 4,000 sequencer: Q30 > 80% indicates good sequencing quality. After quality control, trim the 3/adapter using Cutadapt software (v1.9.3) and remove low-quality reads, and harvest high-quality clean reads. Align a clean read of the input library with a reference genome (GRCh38. Geocoding v32) using STAR software (Dominissini et al., 2016) and identify mRNA peaks using DCC software (Cheng et al., 2016). Next, align clean reads of all libraries with reference genomes using Hisat2 software (v2.0.4) (Kim et al., 2015). Identify m5C peaks on mRNA using MACS software (Zhang et al., 2008). Since m5C antibody pulls down not only a single base, it is theoretically only accurate to the methylation zone, not to distinguish between “2C positions” motif analysis uses sequences of 50 bp before and after peak vertex coordinates. This result reflects the sequence characteristics upstream and downstream of m5C methylation sites, and does not necessarily include the specific single base site where m5C is located. If the methylation region falls on multiple mRNA transcripts, the transcript with the longest overlap between the methylation region and transcript will be selected for annotation. Our annotation of mRNA was manually integrated in several databases, including NCBI, Ensembl and UCSC.In addition, different methylation peaks were identified using DiffReps software (Shen et al., 2013): m5C peaks with a fold change of >2 or <0.5 (*p* < 0.00001) in CRC were considered to be upregulated methylation or downregulated methylation. (Shen et al., 2013).Peaks that overlap exons of protein-coding genes identified using software and m5C sections were selected using internally developed scripts for further annotation.

### 2.5 Statistical analysis

The m5C peak on the mRNA of the two samples in the CRC group was combined to obtain the m5C peak of the CRC group, sh-NSUN2 HCT15 Groups are treated in the same way. Use the bed tool software to find a common peak between two groups. Methylation peak sequences of 50 bp on each side of the vertices were scanned using DREME software (Bailey, 2011) to find meaningful motifs. The E value of the motif is calculated as the enriched *p*-value multiplied by the number of candidate motifs tested, and the enriched *p*-value is calculated using the Fisher exact test to enrich the motif in the positive sequence. The lower the E value of the pattern, the higher its credibility. Subsequently, methylation folded enrichment (FE) of each mRNA in two pairs of samples was collected (Bailey, 2011) and log2 conversion was performed. Log FE values are used for clustering in heatmap.2 packages. In addition, we counted the mRNA regions where the m5C peaks are located in each sample according to the published method (Luo et al., 2014) and plotted the results as a pie chart. (Luo et al., 2014).

### 2.6 Bioinformatics analysis

**FIGURE 1:**
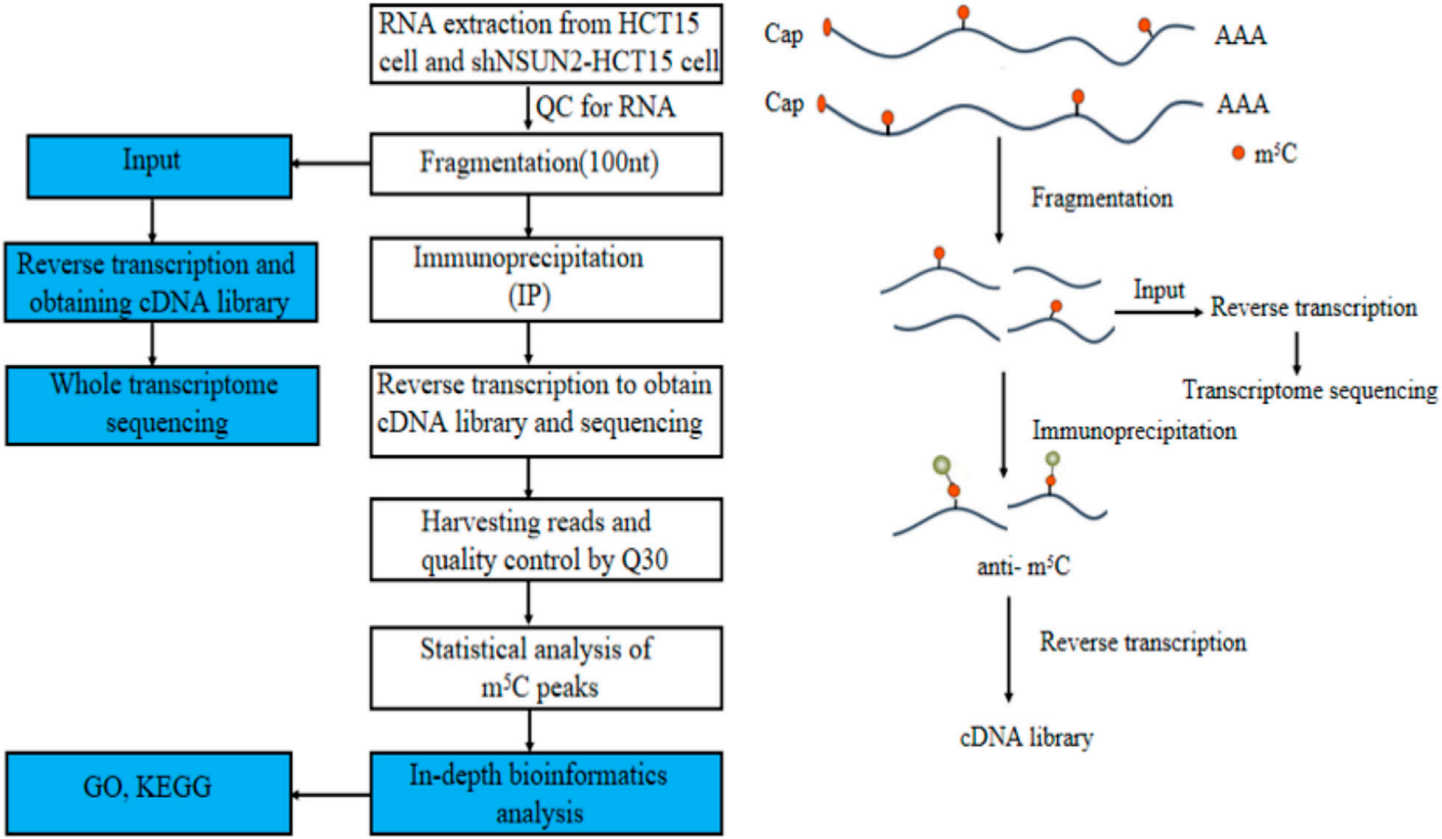
Flowchart of the study.

The Gene Ontology (GO) project is a structured, controlled vocabulary for annotating genes and gene products (Huang et al., 2019) containing three parts: biological processes (BP), molecular function (MF), and cellular components (CC). Differentially methylation genes were used to perform GO function analysis (http://www.geneontology.org) to annotate and speculate on the function of these differential methylated genes. A gene item with a *p*-value of <0.05 was considered statistically significant. Meanwhile, pathway analysis of differential methylated genes was performed using the Kyoto Encyclopedia of Genes and Genomes (KEGG) (https://david.ncifcrf.gov/) to annotate and speculate on pathways they might be involved in. Pathways with a *p*-value of <0.05 are considered significantly enriched. In addition, we used the enrichment intensity fold change value of the two groups of samples in the MeRIP-seq experiment to rank all the coded genes with signals. We chose FDR <0.25 as the screening criterion. Figure 1 is our technology roadmap.

## 3 Results

### 3.1 General features of m5C methylation in HCT15 cell and sh-NSUN2 HCT15 cell

**FIGURE 2:**
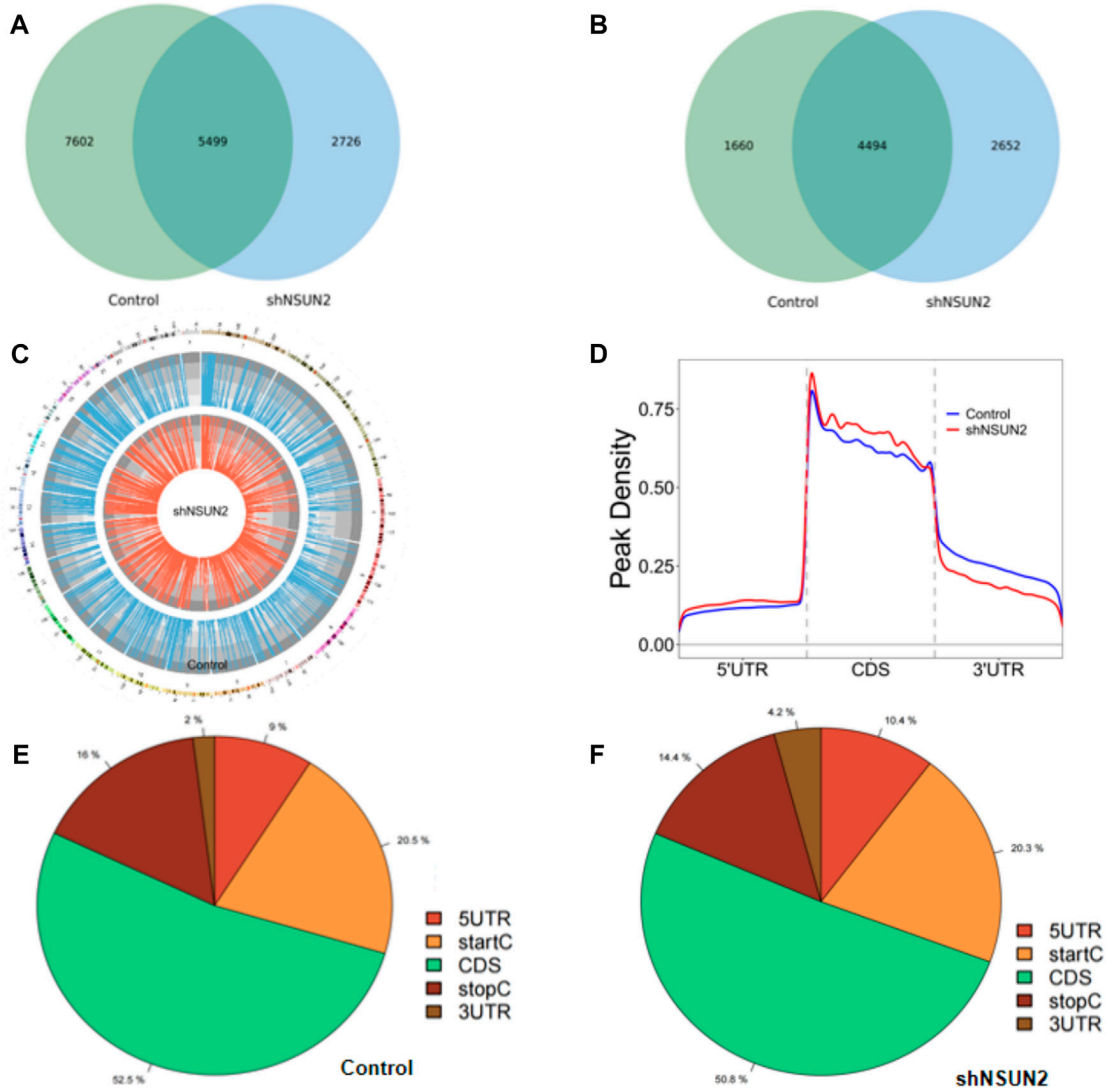
Characteristics of m5C peaks in HCT15 cell and sh-NSUN2 HCT15 cell **(A)** Venn diagram of m5C peaks in HCT15 cell and sh-NSUN2 HCT15 cell. **(B)** Venn diagram of m5C genes in HCT15 cell and sh-NSUN2 HCT15 cell. **(C)** Visualization of m5C at the chromosome level in HCT15 cell and sh-NSUN2 HCT15 cell **(D)** Accumulation of the region of average m5C peaks along all transcripts in HCT15 cell and sh-NSUN2 HCT15 cell. **(E,F)** Pie chart of the source of methylated mRNA in HCT15 cell and sh-NSUN2 HCT15 cell.

In this study, we found 13,101 clean methylation peaks in HCT15 cell and 8,225 clean methylation peaks in sh-NSUN2 HCT15 cell. We mapped up to 6,154 annotated genes of HCT15 and 7,146 annotated genes of sh-NSUN2 HCT15 cell. Among them, 5,499 m5C mRNA peaks appeared in HCT15 cells and sh-NSUN2 HCT15 cells, corresponding to 4,494 annotated genes (Figures 2A,B). We further used Circos software to study the distribution of m5C peaks on chromosomes and found that the number and distribution of m5C peaks on each chromosome of HCT15 cells and sh-NSUN2 HCT15 cells were different, and the difference between chromosome 1 and chromosome 19 was the most obvious (Figure 2C).

Furthermore, analysis of methylation peak sources shows that m5C is distributed in all regions of the mRNA and is predominantly enriched in coding sequences (Figures 2D–F). However, the m5C peak in HCT15 cells and that in sh-NSUN2 HCT15 cells showed different patterns, and with a relative increase in the number of m5C peaks in the coding sequence (CDS) (HCT15: 52.5%, sh-NSUN2 HCT15: 50.8%) and start codon (start C) (HCT15: 20.5%, sh-NSUN2 HCT15: 20.3%), and stop codon (stop C) (HCT15:16%, sh-NSUN2 HCT15:14.4%) and a relative decrease in the 5′untranslated region (5′UTR) (HCT15:9%, sh-NSUN2 HCT15:10.4%) and 3′untranslated region (3′UTR) (HCT15:2%, sh-NSUN2 HCT15: 4.2%).

### 3.2 m5C motif analysis and differential m5C methylation analysis

**FIGURE 3:**
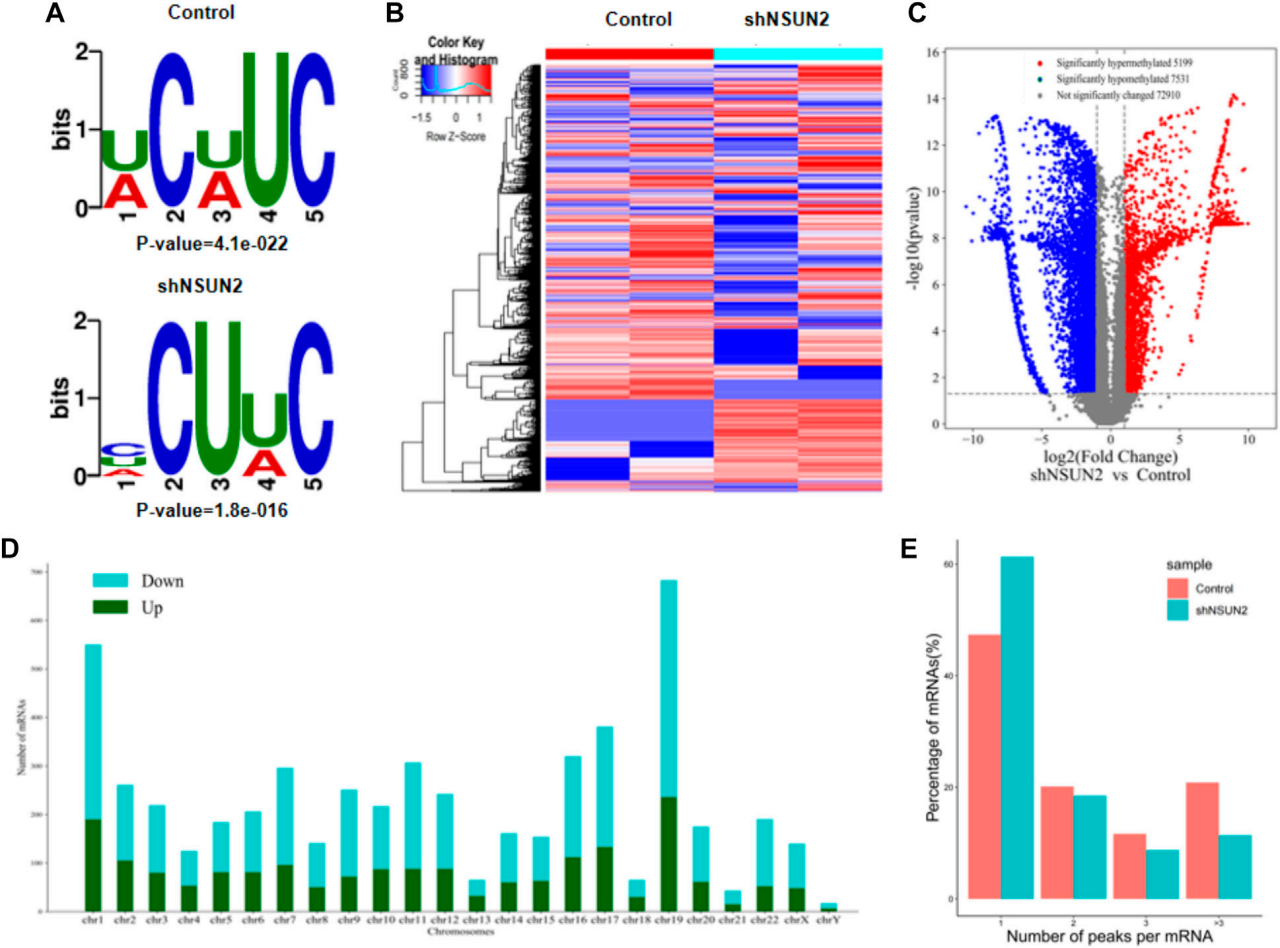
Characteristics of altered m5C peaks in HCT15 cell and sh-NSUN2 HCT15 cell **(A)** The sequence motif of m5C sites in HCT15 cell and sh-NSUN2 HCT15 cell **(B)** Cluster analysis of methylation in HCT15 cell and sh-NSUN2 HCT15 cell. **(C)** Volcano plots showing the significantly differential m5C peaks. **(D)** The distributions of altered m5C peaks in human chromosomes. **(E)** The number of m5C peaks in HCT15 cell and sh-NSUN2 HCT15 cell on each mRNA. Most mRNAs have only one methylation peak.

Our results showed that WCWUC was the most common and reliable methylation site motif (*p* = 1.3e-024) in HCT15 cell, whereas HCUWC was the most common and reliable methylation site motif (*p* = 1.8e-016) in sh-NSUN2 HCT15 cell (Figure 3A). We performed methylation heat map analysis and clustering analysis on the total data. The results of cluster analysis showed that the expression was obvious different between the groups and relatively consistent within the groups. These differences are most likely caused by the decrease in m5C modification levels caused by the absence of NSUN2(Figure 3B). In addition, we identified 5,199 upregulated m5C methylation peaks and 7,531 downregulated m5C methylation peaks using DiffReps software (Figure 3C). We list the top 10 mRNAs with the greatest change in folding in Table 1 -Table 2. The dysregulated m5C methylation peaks are distributed on all chromosomes, especially on chromosome 1 (Figure 3D). In addition, to determine the number of m5C peaks on each mRNA, we counted the methylated peaks and the mRNAs corresponding to the methylated peaks. The results showed that most of the mRNA with methylation sites in sh-NSUN2 HCT15 cells had only one methylation peak (61.33%), while the proportion in HCT15 cells was low (47.35%). Meanwhile, the number of mRNA with 3 or more methylation peaks on one mRNA in HCT15 cells was higher than that in sh-NSUN2 HCT15 cells (*p* < 0.05) (Figure 3E).

**TABLE 1:** Top Ten Upregulated m5C Methylation Peaks.

Chrom	Peak ID	Gene name	Fold change	*p*-value
chr19	diffreps_peak_203849	CACNA1A	995.1	2.40235E-09
chr10	diffreps_peak_46103	SYT15	843.5	1.07187E-11
chr1	diffreps_peak_30367	FMO4	795.4	1.71073E-14
chr7	diffreps_peak_367178	NOD1	762.4	1.22969E-11
chr16	diffreps_peak_156852	HYDIN	756.4	2.41567E-09
chr15	diffreps_peak_138366	GABARAPL3	696	2.38814E-09
chr9	diffreps_peak_417313	TTLL11	643.2	3.08756E-09
chr5	diffreps_peak_329823	DMGDH	629.8	2.38699E-09
chr11	diffreps_peak_67714	PRG2	624.8	3.24616E-09
chr20	diffreps_peak_259197	LBP	616.4	1.58187E-09

**TABLE 2:** Top Ten Downregulated m5C Methylation Peaks.

Chrom	Peak ID	Gene name	Fold change	*p*-value
chr14	diffreps_peak_116180	AL627171.1	1858.4	5.61883E-09
chr6	diffreps_peak_350624	C6orf223	1434.7	4.44192E-10
chr1	diffreps_peak_14335	FOXO6	1078.5	1.34935E-08
chr17	diffreps_peak_181805	TSEN54	1007.2	8.57244E-10
chr20	diffreps_peak_257568	CBFA2T2	751.5	3.1401E-13
chr11	diffreps_peak_61091	AK126380	730.4	1.6988E-10
chr3	diffreps_peak_289417	SHISA5	728	4.26002E-13
chr2	diffreps_peak_249694	ALPI	686.6	2.41449E-09
chr1	diffreps_peak_32830	ASCL5	643.1	8.95643E-09
chr2	diffreps_peak_249868	NGEF	613.1	1.25898E-08

### 3.3 Gene ontology (GO) enrichment analysis

**FIGURE 4:**
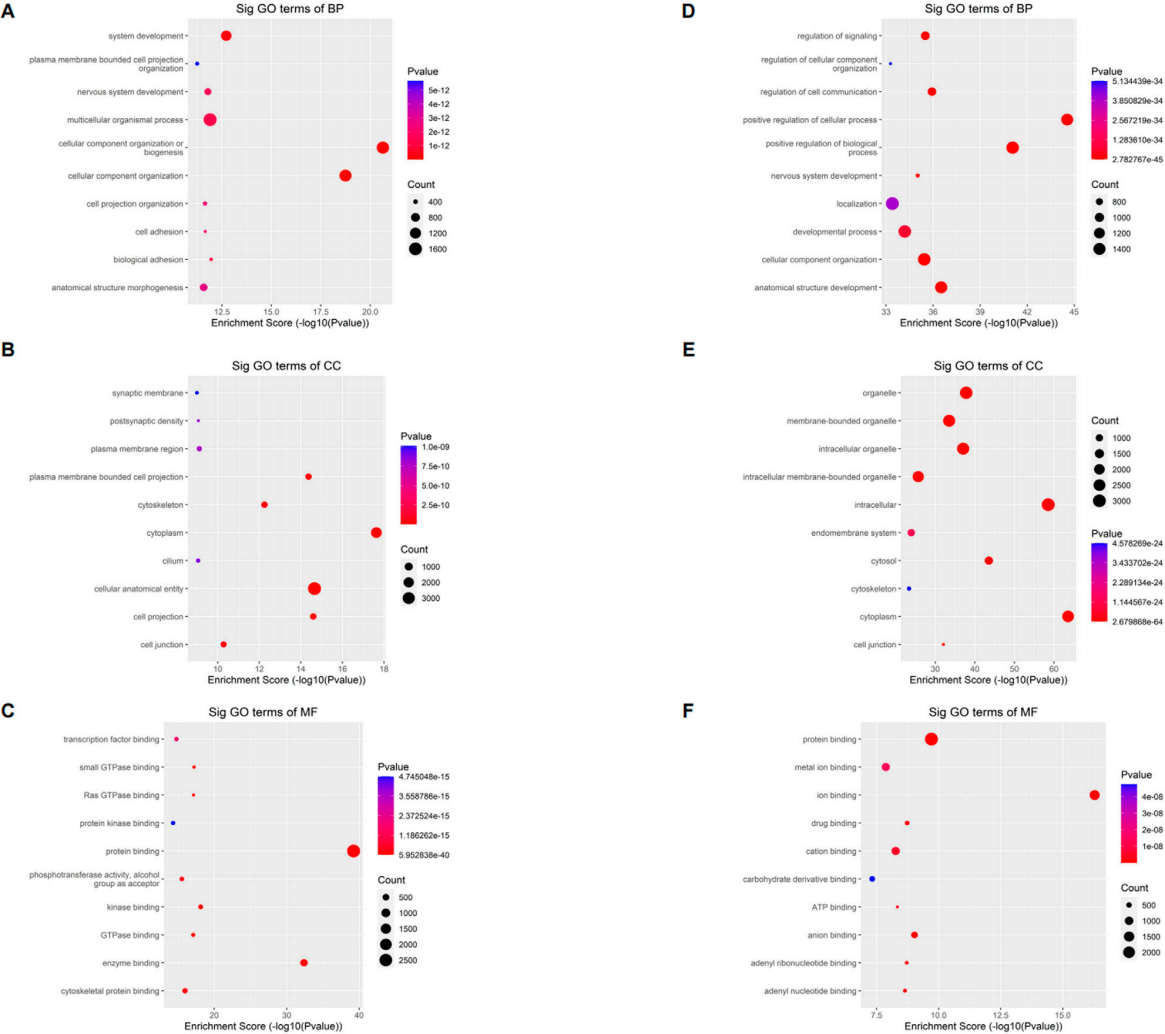
Gene ontology analyses of HCT15 and shNSUN2-HCT15 cell **(A)** Biological processes, **(B)** cellular components, and **(C)** molecular functions of genes annotated by upregulated m5C peaks in sh-NSUN2 HCT15 cell group. **(D)** Biological processes, **(E)** cellular components, and **(F)** molecular functions of genes annotated by downregulated m5C peaks in sh-NSUN2 HCT15 cell group. The top ten most significant terms are shown for each analysis.

The full name of GO database is Gene Ontology. They divided the function of genes into three parts: cellular component (CC), molecular function (MF) and biological process (BP). Using the GO database, we can get what our target genes are mainly related to at the CC, MF and BP levels. To understand the biological function of differentially methylated mRNAs in sh-NSUN2 HCT15 cell and HCT15 cell, we performed GO analyses. For the upper methylated m5C site in sh-NSUN2 HCT15 cells, GO analysis showed that these genes were mainly enriched in system development (GO term: BP), Synaptic membrane (GO term: CC), and transcription factor binding (GO term: MF) (Figures 4A–C). For the m5C site of hypomethylation in sh-NSUN2 HCT15 cells, GO analysis showed that these genes were predominantly enriched in the regulation of signaling (GO term: BP), organelle (GO term: CC), and protein binding (GO term: MF) (Figures 4D–F).

### 3.4 The Kyoto encyclopedia of genes and genomes (KEGG)pathway analysis

**FIGURE 5:**
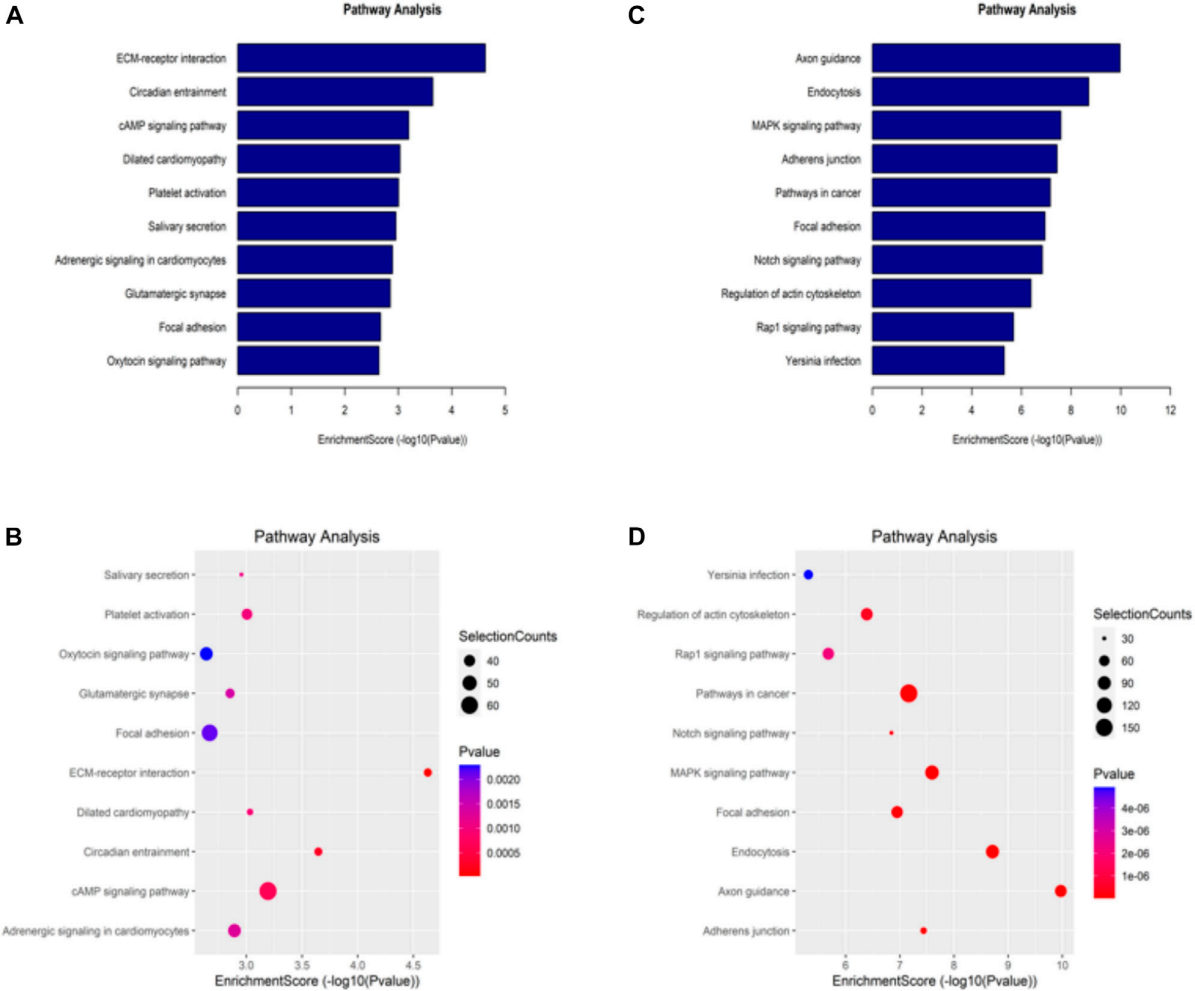
Kyoto Encyclopedia of Genes and Genomes analysis of differentially methylated genes in HCT15 and shNSUN2-HCT15 cell **(A,B)** Pathway analysis of up-methylated genes in sh-NSUN2 HCT15 cell group. **(C,D)** Pathway analysis of down-methylated genes in sh-NSUN2 HCT15 cell group.

The KEGG database is one of the most used bioinformatics databases in the world, known for its “repository of advanced features and utilities for understanding biological systems.” To clarify the biological function of differentially methylated mRNAs in sh-NSUN2 HCT15 cell and HCT15 cell, we performed KEGG Pathway Analysis. KEGG Pathway analysis showed that the upregulation of mRNA in sh-NSUN2 HCT15 cell was mainly involved in the ECM receptor interaction pathway, Circadian entrainment pathway, and cAMP signaling pathway (Figures 5A,B). Down-methylated mRNAs were significantly enriched in Axon guidance, Endocytosis, and MAPK signaling pathway (Figures 5C,D).

## 4 Discussion

As a very important post-transcriptional modification, m5C RNA methylation has been shown to be involved in the occurrence and progression of a variety of cancers. In this study, we first utilized the MeRIP-seq approach to characterize m5C methylomes in HCT15 cell and sh-NSUN2 HCT15 cell. We found 13,101 methylation peaks in HCT15 cell and 8,225 methylation peaks in sh-NSUN2 HCT15 cell. According to relevant literature reports (He et al., 2022), the proportion of m5C/C modifications in human mRNA is approximately between 0.02% and 0.09%. The number of m5C modification peaks detected in this study is also approximately within the above range. The number of m5C modification peaks detected in the other two studies (Zhang et al., 2020; Meng et al., 2021) is also approximately consistent with this study. The research team at Sun Yat-sen University conducted transcriptome sequencing analysis of 7 human tissues and 10 mouse tissues, identifying a total of 3,212 and 2,498 high confidence sites (Huang et al., 2019). They believe that there are significant differences in the number and methylation levels of mRNA m5C sites in different tissues. Given that the number of m5C modification sites detected by us is several times larger than the above studies, we also tend to believe that the reason for the difference in the number of m5C modification sites is that there are significant differences in the degree of m5C modification in different species and tissues.

DirectRMDB (Zhang et al., 2023) and m5C Atlas (Ma et al., 2022) are two public databases that integrate a large number of m5C sequencing results. We compared the predicted m5C modification sites with m5C-Atlas and DirectRMDB to identify novel m5C sites in HCT15/NSUN2KD-HCT15. Here, we use RNF126 (NM_194,460) as an example. We used m5C-Atlas to predict the m5C site of RNF126. The potential m5C site of RNF126 predicted by this database was believed to be located in Chromosome 19:648,944–648948. This result was repeated in 3 cell lines (HCT116, Hela, T24) and bladder cancer and its adjacent tissues. DirectRMDB database are predicting the RNF126 m5C site is 648,944, 8 cell line (ENDOC, H460, HEK293T, HepG2, K562, PDX, SEAC, u87) result supports the prediction. In our study, the potential m5C sites of RNF126 in the HCT15 cell line were considered in Chromosome 19:663046–663233. This is inconsistent with the conclusions of the previous two public databases (Ma et al., 2022; Zhang et al., 2023). This may be the new m5C site of RNF126. However, this may be due to different modification sites of different cell lines or different sequencing methods, which needs to be confirmed by further experimental studies.

Our study found that m5C sites are distributed in all regions of mRNA and occur on all chromosomes, especially the CDS region (HCT15: 52.5%, sh-NSUN2 HCT15: 50.8%) and chromosome 19. According to research (Huang et al., 2019) by Tao Huang et al., the density of the m5C site in CDS is the lowest. Another study (Yang et al., 2017) does not support this view, pointing out that m5C is also abundant in the immediate downstream region of the translation initiation site. Our results show that most of the m5C modification sites are in the CDS region, which is consistent with the results of Xin Yang (Yang et al., 2017). Similarly, another research (Schumann et al., 2020) also support this conclusion. They believe that the m5C site of NSUN2 mediated messenger RNA tends to appear in the C/G-C/G-m5C/A-G-G-G-G-G portion of the primary sequence, and these m5C sites are enriched near the 5′UTR and translation initiation sites.

We identified thousands of methylation peaks in the mRNA and found differences in the number and distribution of methylation peaks between the two groups. The degree of methylation of mRNA in HCT15 was significantly higher than that of sh-NSUN2 HCT15 cells. This may be caused by demethylation of sh-NSUN2 HCT15 cells. In addition, the number of mRNA-mapped methylation peaks in HCT15 cells was significantly higher than in sh-NSUN2 HCT15 cells. The results of cluster analysis showed that the degree of methylation could clearly distinguish HCT15 cells from sh-NSUN2 HCT15 cells, further confirming the potential relationship between m5C and CRC. The fold change in m5C methylation in HCT15 was significantly higher than in sh-NSUN2 HCT15 cells, especially for certain mRNAs (CACNA1A, SYT15, FMO4).

Previous studies have suggested that mRNA has two types of m5C sites (named Type I and Type II sites), which respectively have unique sequence and structural characteristics. The writer protein at Type I site is NSUN2. The Type I site is enriched in most tissue and cell types and is located at the 5′UTR of a stem-ring structure, and the 3′UTR has a guanine-rich (G-rich) motif. The writer protein at the Type II site is NSUN6(Liu et al., 2021). Type II sites showed strong tissue specificity, ranging from 1% to 40%. The Type II site is in the ring region of a stem-ring structure with a TCCA motif at the 3′UTR of the site. In this study, DREME software is used to find that potential new m5C motifs may include: GRAGVAG, GCCGCC, CCDGGC, *etc.*, but these need to be further verified. The motifs analysis showed that the two groups of most reliable motifs were very similar, suggesting that the different motifs in the two sets of m5C may be due to different numbers of methylases rather than methylase classes. A number of studies have shown that the distribution of methylation sites in different regions of mRNA is critical for mRNA stability and regulation of translation. N1-methyladenosine (m^1^A) is significantly enriched near the onset of translation in mammalian and yeast cells, and m1A is associated with higher protein expression (Dominissini et al., 2016; Li et al., 2016).

Recent studies have shown that m5C regulates eukaryotic translation in a negative manner (Delatte et al., 2016; Hoernes et al., 2016), which can also work through the m5C site near the start codon. The results showed that the m5C peak in the initiation codon of HCT15 cells (20.5%) was like that in sh-NSUN2 HCT15 cells (20.3%). Our results show that there are fewer m5C peaks at 3′UTR in HCT15 cells (2%) and sh-NSUN2 HCT15 cells (4.2%), which may also lead to differences in cell function. For example, the m5C of 3′UTR may affect the binding of miRNA or RBP to mRNA, thus regulating the translation process (Squires et al., 2012). Since the mechanism has not been confirmed in CRC, more complex experiments are needed for further verification.

We mapped up to 6,154 annotated genes of in HCT15 and 7,146 annotated genes of sh-NSUN2 HCT15 cell. Of these, 5,499 m5C mRNA peaks were present in both cell lines, corresponding to 4,494 annotated genes. Furthermore, we found that differentially methylated transcripts are enriched in various important cell biology processes, such as system development, Synaptic membrane, and transcription factor binding. Through KEGG analysis, we found the differentially methylated transcripts were enriched in some key cancer related pathways such as ECM receptor interaction pathway, Circadian entrainment pathway, and cAMP signaling pathway. In summary, our data provide a comprehensive picture of m5C methylation in CRC and open another window for understanding the pathogenesis of CRC.

We know that results from 1 cell line are relatively weak to show a consistent m5C profile in colorectal cancer cells. To compensate for this deficiency, we conducted a comprehensive search of m5C studies of colorectal cancer cell lines, we found that the dataset coded GSE74771 contained the Bisulfite-seq data of HCT116, which was included in the m5C-Atlas database and could be used for the prediction of m5C sites. We used m5C-Atlas to predict the m5C site of RNF126 (NM_194,460), and then compared it with our data. We found that the potential m5C site of RNF126 was believed to be located at 648,944–648948 in HCT116 cell line. In HCT15 cell lines, the potential m5C site for RNF126 is thought to be in 663,046–663233. This may be due to different modification sites of different cell lines or different sequencing methods, which needs to be confirmed by further experimental studies. The mutual verification of multiple independent sets of data helps us to better understand the role of m5C modification in colorectal cancer.

In conclusion, our study shows a difference in the number and distribution of RNA m5C peaks between HCT15 cells and sh-NSUN2 HCT15 cells. We further performed bioinformatics analysis to initially reveal the potential direction of m5C modification on CRC regulation at the post-transcriptional level. Targeted regulation of m5C mRNA is a new treatment method to guide the individualized treatment of colorectal cancer patients, but its specific mechanism needs to be further explored.

## Figures and Tables

**Table:** 

**m5C**	5-Methylcytosine
**CRC**	Colorectal cancer
**m6A**	N6-Methyladenosine
**m1A**	N1-Methyladenosine
**GO**	Gene ontology
**KEGG**	Kyoto Encyclopedia of Genes and Genomes
**BP**	Biological processes
**MF**	Molecular functions
**CC**	Cellular components
**IP**	Immunoprecipitation

## Data Availability

The datasets presented in this study can be found in online repositories. The names of the repository/repositories and accession number(s) can be found in the article/Supplementary Material.
