# Supplementary material for: Overview of distinct 5-methylcytosine profiles of messenger RNA in normal and knock-down NSUN2 colorectal cancer cells
Source: Front Genet. 2023 Apr 24;14:1121063. doi: 10.3389/fgene.2023.1121063 (PMC10166136; doi:10.3389/fgene.2023.1121063)
Supplement: Supplementary file 1 [file DataSheet1.ZIP › SupplementaryNSUN21211/Table S1.doc]

**Supplementary data for**

**Overview of distinct 5-methylcytosine profiles of messenger RNA in normal and knock-down NSUN2 colorectal cancer cells**

**Supplementary table 1 Sequences for shRNA**

| **Name** | **Sequences (5’ – 3’)** |
| --- | --- |
| sh-NSUN2 | CACGTGTTCACTAAACCCTAT |
