# Supplementary figures and images for: Overview of distinct 5-methylcytosine profiles of messenger RNA in normal and knock-down NSUN2 colorectal cancer cells
Source: Front Genet. 2023 Apr 24;14:1121063. doi: 10.3389/fgene.2023.1121063 (PMC10166136; doi:10.3389/fgene.2023.1121063)

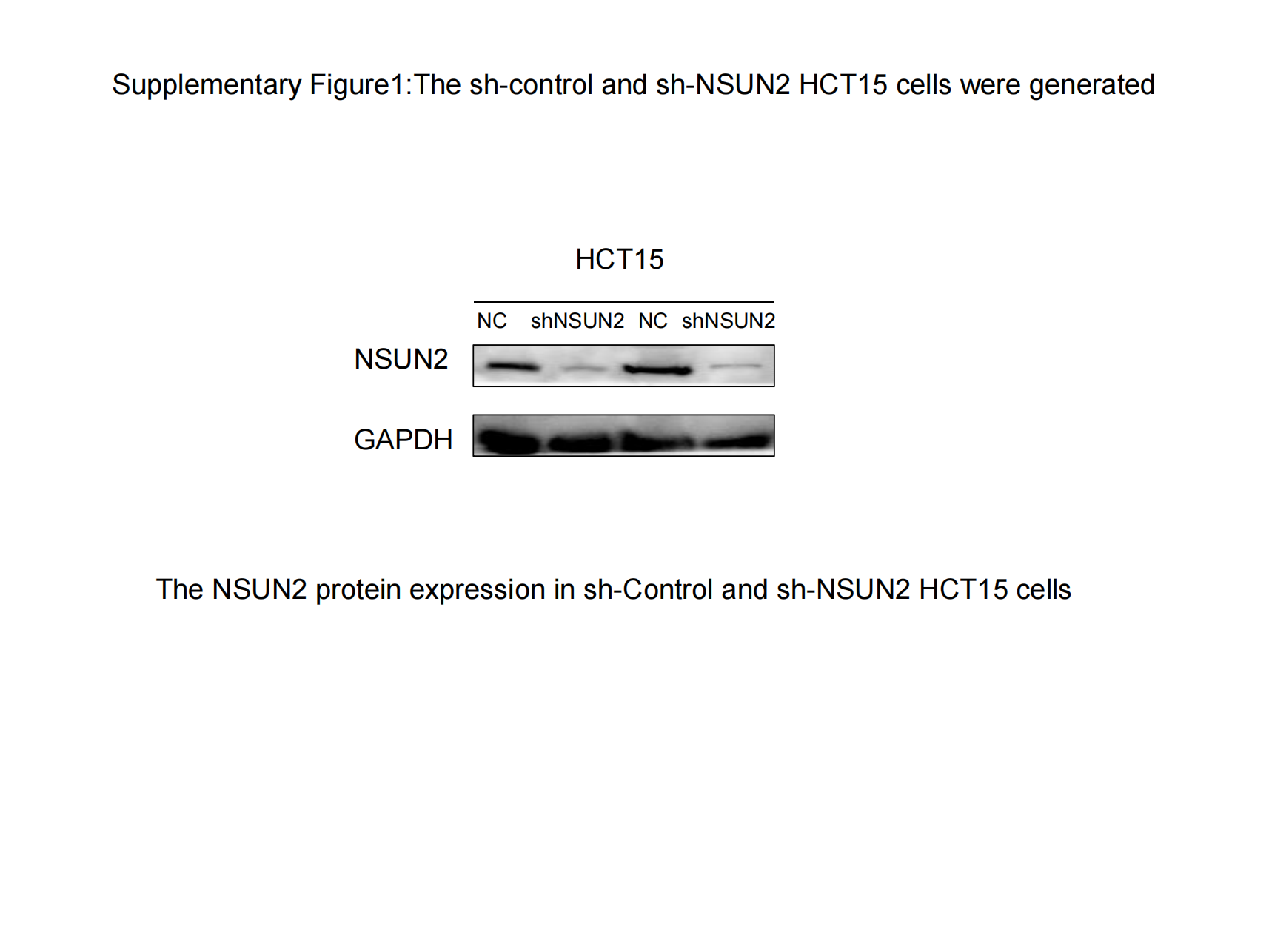

Supplement: Supplementary file 1 [file DataSheet1.ZIP › SupplementaryNSUN21211/Figure S1.tif]

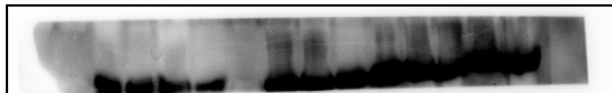

GAPDH

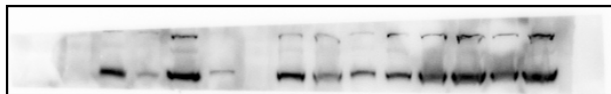

NSUN2

Supplement: Supplementary file 1 [file DataSheet1.ZIP › SupplementaryNSUN21211/full-length gels and blots.pdf]
