# Supplementary material for: Overview of distinct 5-methylcytosine profiles of messenger RNA in normal and knock-down NSUN2 colorectal cancer cells
Source: Front Genet. 2023 Apr 24;14:1121063. doi: 10.3389/fgene.2023.1121063 (PMC10166136; doi:10.3389/fgene.2023.1121063)
Supplement: Supplementary file 1 [file DataSheet1.ZIP › SupplementaryNSUN21211/Table NSUN2.doc]

| **Chrom** | **Peak ID** | **Gene Name** | **Fold change** | **P-value** |
| --- | --- | --- | --- | --- |
| chr19 | diffreps_peak_203849 | CACNA1A | 995.1 | 2.40235E-09 |
| chr10 | diffreps_peak_46103 | SYT15 | 843.5 | 1.07187E-11 |
| chr1 | diffreps_peak_30367 | FMO4 | 795.4 | 1.71073E-14 |
| chr7 | diffreps_peak_367178 | NOD1 | 762.4 | 1.22969E-11 |
| chr16 | diffreps_peak_156852 | HYDIN | 756.4 | 2.41567E-09 |
| chr15 | diffreps_peak_138366 | GABARAPL3 | 696 | 2.38814E-09 |
| chr9 | diffreps_peak_417313 | TTLL11 | 643.2 | 3.08756E-09 |
| chr5 | diffreps_peak_329823 | DMGDH | 629.8 | 2.38699E-09 |
| chr11 | diffreps_peak_67714 | PRG2 | 624.8 | 3.24616E-09 |
| chr20 | diffreps_peak_259197 | LBP | 616.4 | 1.58187E-09 |

Table 1 Top Ten Up regulated m5C Methylation Peaks

Table 2 Top Ten Down regulated m5C Methylation Peaks

| **Chrom** | **Peak ID** | **Gene Name** | **Fold change** | **P-value** |
| --- | --- | --- | --- | --- |
| chr14 | diffreps_peak_116180 | AL627171.1 | 1858.4 | 5.61883E-09 |
| chr6 | diffreps_peak_350624 | C6orf223 | 1434.7 | 4.44192E-10 |
| chr1 | diffreps_peak_14335 | FOXO6 | 1078.5 | 1.34935E-08 |
| chr17 | diffreps_peak_181805 | TSEN54 | 1007.2 | 8.57244E-10 |
| chr20 | diffreps_peak_257568 | CBFA2T2 | 751.5 | 3.1401E-13 |
| chr11 | diffreps_peak_61091 | AK126380 | 730.4 | 1.6988E-10 |
| chr3 | diffreps_peak_289417 | SHISA5 | 728 | 4.26002E-13 |
| chr2 | diffreps_peak_249694 | ALPI | 686.6 | 2.41449E-09 |
| chr1 | diffreps_peak_32830 | ASCL5 | 643.1 | 8.95643E-09 |
| chr2 | diffreps_peak_249868 | NGEF | 613.1 | 1.25898E-08 |
